# Risk of cancer among HIV-infected patients from a population-based nested case–control study: implications for cancer prevention

**DOI:** 10.1186/s12885-015-1099-y

**Published:** 2015-03-16

**Authors:** Chang-Hua Chen, Chih-Yuan Chung, Li-Hsuan Wang, Che Lin, Hsiu-Li Lin, Hsiu-Chen Lin

**Affiliations:** 1 Division of Infectious Diseases, Department of Internal Medicine, Changhua Christian Hospital, Changhua, Taiwan; 2 College of Medicine & Nursing, Hung Kuang University, Taichung City, Taiwan; 3 Division of Hematology and Oncology, Department of Internal Medicine, Changhua Christian Hospital, Changhua, Taiwan; 4 Department of Pharmacy, Taipei Medical University Hospital, Taipei, Taiwan; 5 School of Pharmacy, College of Pharmacy, Taipei Medical University, Taipei, Taiwan; 6 Comprehensive Breast Cancer Center, Changhua Christian Hospital, Changhua, Taiwan; 7 Department of Environmental Engineering, National Chung-Hsing University, Taichung, Taiwan; 8 Graduate Institute of Biomedical Informatics, College of Medical Science and Technology, Taipei Medical University, Taipei, Taiwan; 9 Department of Neurology, General Cathay Hospital, Sijhih Branch, New Taipei City, Taiwan; 10 Department of Pediatrics, School of Medicine, College of Medicine, Taipei Medical University, No. 250 Wu-Hsing Street, 11031 Taipei, Taiwan; 11 Department of Laboratory Medicine, Taipei Medical University Hospital, Taipei, Taiwan

**Keywords:** Cancer, Human immunodeficiency virus, Taiwan, Risk

## Abstract

**Background:**

The burden of cancer is likely to increase among the human immunodeficiency virus (HIV)-positive population as it ages due to successful antiretroviral therapy (ART). The purpose of this study was to determine the risk of cancer in HIV-infected patients.

**Methods:**

This study was a matched nested case–control study. It was performed using the National Health Insurance Research Database of Taiwan. The control group included non–HIV-infected patients matched by sex, age, and year of enrollment. Logistic regression analyses were performed and simultaneously adjusted for potential confounders (income, urbanization, and Charslon index of comorbidity to evaluate HIV infection as an independent risk of cancer. We calculated the overall and sex-specific standardized incidence ratios (SIR) to investigate the pattern of cancer risk and overall cancer risk in the patients with HIV infection.

**Results:**

Of the 1,115 HIV-infected patients, 104 (9.33%) developed cancer during the 11-year follow-up period. The risk of cancer for patients with HIV infection was significant (adjusted odds ratio = 3.89, 95% confidence interval [CI] = 2.92–5.19) after adjustment for potential confounders. There was a significantly increased risk of developing non-Hodgkin lymphoma (SIR = 25.73, 95% CI = 6.83-90.85), cervical cancer (SIR = 4.01, 95% CI = 1.0-16.06), lymphoma (SIR = 20.26, 95% CI = 5.86-70.10), and respiratory and intrathoracic cancer (SIR = 20.09, 95% CI = 2.34-172.09) compared with the control group. In addition, HIV-infected patients were at significant risk for renal, oral, breast, liver, skin, and colorectal cancer.

**Conclusions:**

Patients with HIV infection are at increased risk for several specific cancers. Our results support the implementation of an active and accelerated cancer screening schedule for patients with HIV infection to increase their life span.

## Background

After use of combined antiretroviral therapy (cART), highly active antiretroviral therapy has dramatically improved the survival of human immunodeficiency virus (HIV)-infected patients [1]. The substantial improvement in survival after HIV infection has led to an increasing clinical impact of long-term morbidities, including cancers, in this population. An increased risk of cancer has been recognized in HIV-infected patients since the beginning of the HIV epidemic [2,3]. This is believed to be a result of HIV-induced immune suppression hindering the control of cancer-associated viruses as well as direct effects of HIV replication [4].

The incidence of acquired immunodeficiency syndrome (AIDS)-defining cancers (ADCs) and non-AIDS-defining cancers (NADCs) in HIV-infected patients has increased, but the association between HIV infection and the risk of cancer is both clinically and epidemiologically controversial [1,5]. However, the burden of cancer, particularly NADCs, is likely to increase as HIV-infected patients live longer [5]. Non-AIDS-related causes of morbidity and mortality are becoming increasingly prevalent in HIV-infected patients, and NADCs are emerging as a significant source of mortality [6].

The rising incidence of cancer is a problem throughout the world, including Taiwan. A cancer screening protocol was implemented for the general population of Taiwan by the Bureau of Health Promotion, Department of Health, in 2010. Four types of cancers were targeted: colorectal, breast, oral, and cervical. However, the prevalence of cancer in HIV-infected patients is still uncertain, and the recommendations of a screening protocol for HIV-infected patients are still being debated. The purpose of this study was to elucidate the risk of cancer in patients with HIV infection during the follow-up period after diagnosis compared with patients without HIV infection during the same period. We hope to apply the findings of this study to a cancer prevention protocol for the HIV-infected population.

## Methods

### Data collection

This matched nested case–control study was performed using the National Health Insurance (NHI) Research Database of Taiwan. The data set was obtained from the Longitudinal Health Insurance Database (LHID) 2000 of the NHI program, which was released by the Taiwan National Health Research Institutes (NHRI) in 2013. The NHI program started in Taiwan in 1996 and provides medical care for more than 99% of residents. For the LHID 2000, the NHRI took a random sample of 1 million (4.2%) of the 23,753,407 people enrolled in the NHI program and determined that there were no statistically significant differences in age, sex, and area distribution between the subjects in the sampled group and the original population. The LHID 2000 is a nationwide population-based data set that provides outpatient and inpatient claims for 11 years of follow-up (since 2000) and therefore is an excellent resource for evaluating the risk of cancer in patients with HIV infection. Because the LHID data set consists of de-identified and secondary data released to the public for research, the study was exempt from full review by the Institutional Review Board of Taipei Medical University.

### Study population/case definition

We included data for all inpatients and outpatients with HIV infection who received a diagnosis with the International Classification of Diseases, Ninth Revision (ICD-9-CM) code of 042 and filed claims, means the patients were diagnosed to have HIV infection, between January 2000 and December 2011. This database was constructed with 1,000,000 persons derived from Taiwan’s 23,753,407 citizens. The period of follow-up was from 2000 to 2011. Only the study participants alive at the start of follow up in 2000 were included in the study, and they were followed for 11 years. However, the study participants (including HIV patients and controls) were censored when cancer developed, death occurred, or retrieval from the National Health Insurance system. Our study included 1,115 patients with HIV infection. The control group was selected from the remaining people in the LHID 2000. Because the participants with HIV infection were distributed in a particular sex and age group, we randomly selected participants without HIV infection for the control group (4 controls for each HIV-infected participant) matched to the case group by sex, age, and year of enrollment (year of HIV diagnosis).

We defined a participant with any type of cancer as having a diagnosis with ICD-9-CM codes 140–208 except 166–169 and 177–178. Because the NHI Research Database includes all medical service utilization for all enrollees, we followed up all sampled participants and the matched controls throughout the study period from 2000 to 2011. The stratification of urbanization was based on the research of Liu et al., in which smaller numbers represent more urbanization [7].

The Charlson index of comorbidity was used to adjust the comorbidities of these 2 groups. The comorbidity index has been widely used for risk adjustment in chronic diseases [8], and a list of 19 comorbidities was used to produce a weighted index account for morbidity and mortality rates. HIV/AIDS and malignancy were included in the Charlson index and were the variables of interest in our study; therefore, we used the modified Charlson index, which excluded these 2 diseases.

### Statistical analysis

The SAS 9.1 statistical package (SAS Institute Inc., Cary, NC, USA) was used to perform all analyses in this study. The Pearson chi-square test was used to examine the differences in sociodemographic characteristics (monthly income and urbanization) and potential confounders in the modified Charlson index. Multiple logistic regression analyses were performed and simultaneously adjusted for the potential confounders of income and urbanization, and the Charlson index was used to evaluate the independent risk of cancer with HIV infection.

We calculated the overall and sex-specific standardized incidence ratios (SIRs) to investigate the pattern of cancer risk and overall cancer risk in the patients with HIV infection. The SIRs were calculated by dividing the actual observed number of cancer cases that occurred in the patients with HIV infection by the expected number of cancer cases. The expected number of cancer cases was obtained from the number of cancer cases that would occur in comparison group that corresponded to the age-, gender-, and year of enrollment of HIV-infected participants. Based on the Taiwan population census and National Cancer Registry cancer registry data, we obtained each age and gender strata and the corresponding stratum-specific incidence rates of cancers for the entire population, respectively. We indicated “<0.5” if the case number or expected number was less than 0.5 and “N/A” with no calculation of SIR. The patient developing more than one type of cancer was categorized to each type of cancer. The patient with two occurrences of the same type of cancer during the follow-up period was calculated only once in the SIR. Crude and adjusted odds ratios (ORs) are presented with 95% confidence intervals (CIs). A two-sided *P* value ≤0.05 was considered statistically significant.

## Results

**Figure 1:**
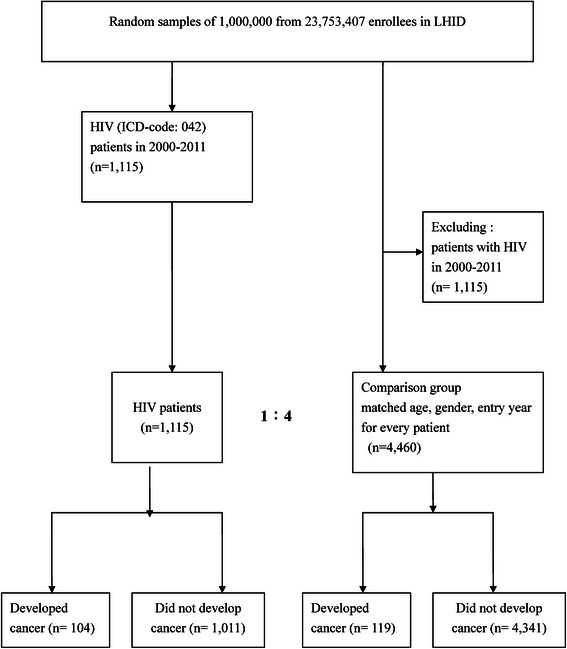
**Study flow chart showing the selection of subjects.** ICD-9-CM: International Classification of Diseases, Ninth revision. HIV: human immunodeficiency virus. LHID: longitudinal health insurance database.

Figure 1 is a flow chart of the participants enrolled in the study, and Table 1 shows the demographic characteristics of the participants with HIV infection and the control group. The ratio of male to female participants was about 3.0. The HIV-infected participants had lower monthly income, less urbanization, and more comorbidities (higher modified Charlson score) than those in the control group. We calculated the mean follow-up times of the study participants after enrollment. They were 55.53 months and 56.12 months, for the HIV-infected patients and controls, respectively. The results are shown in Table 1.

**Table 1:** **Baseline characteristics of the HIV group and the comparison group**

Variable	HIV Patients (*n* = 1,115)	Control (*n* = 4,460)	
	No. (%)	No. (%)	*P-value*
Age			1
≦24	137 (12.3)	548 (12.3)	
25 – 44	626 (56.1)	2,504 (56.1)	
45 – 64	202 (18.1)	808 (18.1)	
≧65	150 (13.5)	600 (13.5)	
Gender			1
Male	836 (75)	3,344 (75)	
Female	279 (25)	1,116 (25)	
Monthly income			<0.001
NTD 0 ~ 17280	502 (45)	1,355 (30.4)	
NTD 17281 ~ 28800	399 (35.8)	1,624 (36.4)	
NTD ≥28801	279 (19.2)	1481 (33.2)	
Urbanization level			<0.001
1	355 (31.8)	1,291 (29)	
2	313 (28.1)	1,412 (31.7)	
3	210 (18.8)	891 (20)	
4	139 (12.5)	538 (12.1)	
5	22 (2)	78 (1.8)	
6	35 (3.1)	126 (2.8)	
7	41 (3.7)	124 (2.8)	
Modified Charlson score			<0.001
0	29 (2.6)	1,644 (36.8)	
1-5	1011 (90.7)	2638 (59.2)	
≧6	75 (6.7)	184 (4.1)	
Mean follow-up time (months)	55.53	56.12	
SD	38.70	36.96	
Q1	26.73	26.9	
Q3	76.98	73.73	
Cancer	104 (9.3)	119 (2.7)	<0.001

Modified Charlson index: Charlson index excluding HIV/AIDS, malignancy.

SD: standard deviation.

Q1: first interquartile, Q3: third interquartile.

The numbers of patients were diagnosed in each year of enrollment were shown in Table 2. We finally enrolled 1,115 patients, of which 99.28% (1107) had received antiretroviral therapy.” Of the 1,115 patients with HIV infection, 104 (9.33%) developed cancer after diagnosis of HIV infection during the follow-up period. The number of subjects who were diagnosed with HIV at the same time as the cancer was 48. The mean and range of duration of infection (from HIV diagnosis to cancer diagnosis) for the remainders was 41.94 (SD = ±35.53) months. In fact, the subjects may have had the HIV infection for a while prior to diagnosis, hence this time would be an underestimate. Table 3 shows that age was a significant risk factor for developing cancer and the risk gradually increased with older age (≥65 years old; adjusted OR = 32.13) after adjustment for multiple confounders. Furthermore, male gender was more significantly associated with cancer than female gender (adjusted OR = 1.45, 95% CI = 1.06–1.99) after adjustment for other risk factors, and a highly modified Charlson score was significantly associated with cancer compared with a low Charlson score (adjusted OR = 7.66, 95% CI = 4.83–13.4) after adjustment. However, the risk of cancer for patients with HIV infection was significant (adjusted OR = 3.89, 95% CI = 2.92–5.19) after adjustment for the previously described confounders, such as age, sex, monthly income, urbanization, and modified Charlson score.

**Table 2:** **The year of enrollment of HIV-infection patients**

Year	Numbers of patients diagnosed with HIV-infection
2000	79
2001	49
2002	52
2003	42
2004	60
2005	97
2006	330
2007	102
2008	92
2009	72
2010	57
2011	83
Total	1,115

**Table 3:** **Logistic regression and multivariate adjusted odds ratios of the cancer risk with different variables**

Variable	Crude OR (95% CI)	Adjusted OR^a^(95% CI)
**HIV**		
No	1	1
Yes	3.75 (2.86 ~ 4.93)**	3.89 (2.92 ~ 5.19)**
Age		
≦24	1	1
25 – 44	3.44 (1.25 ~ 9.49)**	3.14 (1.12 ~ 8.83)*
45 – 64	12.09 (4.39 ~ 33.33)**	11.85 (4.18 ~ 35.57)**
≧65	23.21 (8.48 ~ 63.55)**	32.13 (10.78 ~ 95.75)**
Gender		
Female	1	1
Male	0.72 (0.54 ~ 1.96)	1.45 (1.06 ~ 1.99)*
Monthly income		
NTD 0 ~ 17280	1	1
NTD 17281 ~ 28800	0.54 (0.40 ~ 0.74)**	0.85 (0.60 ~ 1.19)
NTD ≥28801	0.35 (0.24 ~ 0.50)**	0.90 (0.58 ~ 1.41)
Urbanization level		
1	1	1
2	1.23 (0.86 ~ 1.77)	1.11 (0.76 ~ 1.63)
3	1.05 (0.69 ~ 1.61)	0.93(0.59 ~ 1.45)
4	1.85 (1.22 ~ 2.82)**	1.44 (0.92 ~ 2.26)
5	0.91 (0.28 ~ 2.97)	0.46 (0.13 ~ 1.64)
6	1.14 (0.48 ~ 2.69)	0.63 (0.24 ~ 1.62)
7	2.52 (1.35 ~ 4.73)**	1.58 (0.79 ~ 3.18)
Modified Charlson score		
0	1	1
1 ~ 5	2.63 (2.32 ~ 9.08)**	1.73 (1.45 ~ 3.62)**
≧6	11.96 (6.92 ~ 20.67)**	7.66 (4.83 ~ 13.4)**

*P < 0.05, **P < 0.001.

^a^ Adjusted with age, gender, income, urbanization, modified Charlson index.

We further analyzed the site-specific risk of cancer between patients with HIV infection and those without HIV infection (Table 4). Among the ADCs, non-Hodgkin lymphoma (SIR = 25.73, 95% CI = 6.83-90.85), and cervical cancer (SIR = 4.01, 95% CI = 1.0-16.06) show significant increase of the SIR between those with HIV infection and comparison group. Lymphoma (SIR = 20.26, 95% CI = 5.86-70.10), and lung cancer (SIR = 3.49, 95% CI = 1.66-7.37) show significant increase of SIR between those with HIV infection and comparison group. We examined SIR of cancer within the follow-up period and found that renal (SIR = 4.82, 95% CI = 1.47-15.83), oral cancer (SIR = 3.29, 95% CI = 1.36-7.96), breast cancer (SIR = 4.01, 95% CI = 1.16-13.89), liver cancer (SIR = 3.75, 95% CI = 1.76-7.99), skin cancer (SIR = 3.44, 95% CI = 1.15-10.26), and colorectal cancer (SIR = 2.21, 95% CI = 1.06-4.63) were significantly increased risk between those with HIV infection and comparison group. The duration of infection was different by cancer site, the longest duration was cancers of bone and connective tissue (42.44 ± 39.51 months), the shortest duration was lymphoma (17.91 ± 18.17 months). There were 25 and 35 patients had multiple cancers in the HIV-infection and non-HIV groups, respectively.

**Table 4:** **SIR of site-specific cancer of the HIV-infection patients group in different gender and overall population**

	Men			Women			All		
	Cases of cancer			Cases of cancer			Cases of cancer		
Cancer site	Observed	Expected	SIR (95% CI)	Observed	Expected	SIR (95% CI)	Observed	Expected	SIR (95% CI)
**ENT (140–149)**	10	4	3.15*** (1.38 ~ 7.20)	1	<0.5	N/A	11	4	2.77*** (1.28 ~ 5.98))
Oral (140–146)	8	2.75	4.09*** (1.53 ~ 10.93)	1	<0.5	N/A	9	2.75	3.29*** (1.36 ~ 7.96)
Non- Oral (147–149)	2	2.25	0.85 (0.27 ~ 6.72)	<0.5	<0.5	N/A	2	2.25	0.89 (0.19 ~ 4.12)
**GI (150 ~ 159)**	20	7	2.99*** (1.69 ~ 5.27)	10	5	1.96 (0.77 ~ 3.41)	30	12	2.54*** (1.60 ~ 4.06)
Liver (155)	11	3	3.76*** (1.65 ~ 8.55)	2	1.5	1.15 (0.35 ~ 2.43)	13	4.5	3.75*** (1.76 ~ 7.99)
Colorectal (153–154)	7	2.5	2.59* (1.06 ~ 6.72)	4	2.5	1.52 (0.48 ~ 4.82)	11	5	2.21* (1.06 ~ 4.63)
Others (150–152,156-159)	5	1.75	2.91*** (1.92 ~ 9.19)	3	2.25	1.36 (0.75 ~ 3.01)	8	4	2.01 (0.86 ~ 4.70)
Anus (154.2-154.8)	<0.5	<0.5	N/A	1	<0.5	N/A	1	<0.5	N/A
Stomach (151)	2	0.75	2.71 (0.45 ~ 16.24)	1	<0.5	N/A	3	1	3.07 (0.69 ~ 13.73)
**Respiratory & intrathoracic (160–165)**	12	3.25	3.28*** (1.53 ~ 7.03)	4	1	4.53*** (1.21 ~ 14.69)	16	3.75	4.31*** (2.13 ~ 8.75)
Lung (162)	9	3.25	2.83*** (1.21 ~ 6.64)	4	0.5	6.13*** (1.87 ~ 28.69)	13	3.75	3.49*** (1.66 ~ 7.37)
Non Lung (160–161, 163–165)	4	<0.5	N/A	1	<0.5	N/A	5	<0.5	N/A
**Bone,connective tissue (170–176)**	15	<0.5	N/A	7	2.75	3.26*** (1.59 ~ 11.46)	22	3	7.46*** (3.68 ~ 15.12)
Breast (174–175)	1	<0.5	N/A	4	1.25	2.80*** (1.29 ~ 10.01)	5	1.25	4.01* (1.16 ~ 13.89)
Skin & soft tissue (170–173)	2	<0.5	N/A-	4	1.5	8.44*** (1.54 ~ 46.32)	6	1.75	3.44* (1.15 ~ 10.26)
Malignant melanoma of skin (172)	<0.5	<0.5	N/A	<0.5	<0.5	N/A	<0.5	<0.5	N/A
Other skin & soft tissue (170, 171, 173)	2	<0.5	N/A	4	1.5	8.44*** (1.54 ~ 46.32)	6	1.75	3.44* (1.15 ~ 10.26)
Kaposi (176)	12	<0.5	N/A	<0.5	<0.5	N/A	12	<0.5	N/A
Bone (170)	<0.5	<0.5	N/A	<0.5	<0.5	N/A	<0.5	<0.5	N/A
**GU (180–189)**	7	2.5	2.57*** (1.29 ~ 9.88)	9	3	3.71*** (1.84 ~ 12.59)	16	5.5	2.94*** (1.54 ~ 5.61)
Kidney & bladder (188–189)	4	0.5	8.15*** (1.49 ~ 44.58)	2	0.75	2.15 (0.87 ~ 8.65)	6	1.25	4.82*** (1.47 ~ 15.83)
Cervical (180)	<0.5	<0.5	N/A	4	1	4.01* (1.0 ~ 16.06)	4	1	4.01* (1.0 ~ 16.06)
Others (179,181-187)	4	1.25	2.71 (0.76 ~ 9.64)	5	2.5	2.75*** (1.21 ~ 6.25)	9	3.75	2.41* (1.05 ~ 5.53)
**Lymphoma (200–203)**	12	0.75	12.22*** (3.08 ~ 36.12)	3	0.89	3.38(0.63 ~ 8.28)	15	0.75	20.26*** (5.86 ~ 70.10)
Non Hodgkin lymphoma (200, 202.0-202.2, 202.8-202.9)	10	0.5	21.65*** (5.65 ~ 88.75)	3	0.77	3.90(0.73 ~ 9.55)	13	0.5	25.73*** (6.83 ~ 90.85)
**Leukemia (204–208)**	1	1	1.00 (0.10 ~ 8.96)	<0.5	<0.5	N/A	1	1	1.00 (0.10 ~ 8.96)
**CNS (190–192)**	2	<0.5	N/A	3	<0.5	N/A	5	<0.5	N/A
**Thyroid (193)**	<0.5	<0.5	N/A	2	<0.5	N/A	2	1	2.01 (0.37 ~ 10.94)
**Others (194–199)**	6	4	2.05 (0.76 ~ 3.99)	6	3.25	1.76 (0.89 ~ 6.16)	12	7.25	1.66 (0.85 ~ 3.37)

SIR: standardized incidence ratios.

ENT: ear-nasal cavity-throat.

CNS: central nervous system.

GI: gastroenteral.

GU: genitouro.

*P < 0.05, ***P < 0.001.

N/A: no calculation of SIR due to case number or expected number less than 0.5.

Although the risk of occurrence of Kaposi’s sarcoma cannot be calculated by SIR due to the 0% occurrence in the control group, the association of Kaposi’s sarcoma with HIV infection was very strong (n = 12).

## Discussion

To the best of our knowledge, this is a first nationwide, population-based, follow-up study showing that HIV-infected patients are at significant risk for many types of cancers, such as oral cancer, gastrointestinal cancer, respiratory and intrathoracic cancer, bone and connective tissue cancer, genitourinary cancer, lymphoma, and central nervous system cancer. Although our control group was age- and sex-matched with the case group, our study also showed that age and sex were independent risk factors for cancer after adjustment for HIV infection.

Our results showed that there is a significantly higher rate of NADCs occurring in HIV-infected versus non–HIV-infected patients. Our findings are in agreement with those of a study by Cooley, which showed an increase of NADCs in HIV-infected patients [9]. Furthermore, we demonstrated the increased occurrence of many specific cancers in HIV-infected patients. HIV-infected patients have an elevated risk for certain NADCs [1], which is largely attributable to loss of control of the oncogene group and a high prevalence of exposure to other carcinogens [10]. It is possible that either acceleration of carcinogenesis by HIV infection or earlier exposure to risk factors for cancer play a role [1]. The mechanism underlying the association between HIV infection and cancer is still unclear.

Breast cancer is one of the most common malignancies in women in the general population [11]. A large registry linkage study of cART-era data showed that the risk of breast cancer in HIV-infected women now mirrors that of the general population [12]. Studies from different populations or health care systems have provided inconsistent findings [9,11,12], and the data on the incidence and prognosis of breast cancer in HIV-infected patients are limited and conflicting [13-15]. One study reported on the possible mechanisms of environmental pollution and breast cancer [16], and HIV-infected patients seem to be vulnerable to these mechanisms. There may be multiple factors related to the development of breast cancer in these patients, such as low income, low socioeconomic status (SES), and poor lifestyle. However, our data show an increased risk for breast cancer in those with HIV infection. A recent nested case–control study from the Women’s Interagency HIV Study and HIV Epidemiology Research Study cohorts showed that a low risk of breast cancer may be linked to infection with CXCR-4 tropic virus [17]. This demonstrated lower risk was observed in the pre-cART era but not in the cART era [18]. It may explain why our HIV-infected patients, who decreasing CXCR-4 protective effect after using cART, had higher incidence of breast cancer. Future studies may be needed to explore it. Patients in Taiwan with breast cancer are younger (median age of 45 years) than those in Western countries [19,20], and the molecular subtypes of breast cancer in Taiwan differ from those in Western countries [21]. Nevertheless, our findings should remind HIV-infected patients, especially young patients, to examine their breasts regularly for the possibility of breast cancer.

Skin cancers, in particular basal cell carcinoma, are the most common malignancies in the general population [22]. In our study, the SIR of skin cancer indicated a significantly increased risk in those with HIV infection compared with the control group. HIV-infected patients seem to be vulnerable to skin cancer, and this may be attributed to multiple factors, such as high exposure to ultraviolet rays, low SES, and poor lifestyle. Nevertheless, our data support an increased risk for skin cancer in those with HIV infection.

Lung cancer is the most common cause of cancer mortality worldwide [23] and is the third most common malignancy in HIV-infected patients [1,24,25], but lung cancer in the Swiss HIV Cohort Study did not seem to be clearly associated with immunodeficiency or AIDS-related pulmonary disease [26]. In our study, the SIR of lung cancer indicated a significantly increased risk between HIV-infected and non–HIV-infected patients; HIV-infected patients seem to be vulnerable to lung cancer, and this may be attributed to multiple factors, such as smoking, air pollution, low SES, and poor lifestyle. However, our data support an increased risk of lung cancer in those with HIV infection.

In Taiwan, oral cavity cancer was the fifth most prevalent cancer in 2008 [27]. The risk factors for oral cancer include smoking, alcohol consumption, race, chewing of betel leaves and areca nuts, and low SES [28]. HIV RNA and human papillomavirus (HPV) DNA are present in the oral cavity of HIV-positive patients, and the virus-related pathology has been studied [29]. Chaiyachati reported on 2 HIV-infected patients who first developed HPV-related anal squamous cell carcinoma and later oral squamous cell carcinoma [30]. Our study showed that HIV-infected patients have a significant risk of developing oral cancer and cervical cancer. It is possible that oral sex could play an important role in explaining these results in addition to the viral biological effect. The prevention of oral cancer is an integral part of our national cancer-control programs, and we must ensure that HIV-infected patients undergoing screening. If a clinician knows a patient with HIV infection, he/she is more likely to look for AIDS-defining cancers according to the guidelines in 2014 [31].

Our study showed that HIV-infected patients are at significant risk for developing cancer of the gastrointestinal tract. This type of cancer is one type of NADC with various malignancies, included colorectal cancer and liver cancer [9,32,33]. However, the existing data suggest that colorectal cancer develops at a younger age, at an advanced stage, and with an unfavorable prognosis in the HIV population [28]. There have also been reports of various etiologic agents, including genetic agents, immunologic agents, and viruses such as HPV, playing a role in the development of colorectal cancer in this HIV-infected population, which is different from the general population [34,35]. Liver cancer is the fifth most common cancer worldwide [36], and chronic hepatitis C and hepatitis B virus infections are the main risk factors for development of liver cancer. HIV coinfection has been shown to increase the progression of liver fibrosis [37,38] and is likely to eventually increase the incidence of the tumor [33]. Anal cancer, cervical cancer, and liver cancer are each associated with coinfections (i.e., HPV for anal cancer and cervical cancer, and hepatitis B and C viruses for liver cancer), and the risks of these cancers in HIV-infected individuals are increased because of a greater prevalence of coinfection (for HPV and the hepatitis viruses) and an inability to control these infections with a suppressed immune system [39]. Our data support an increased risk of colorectal cancer and liver cancer in those with HIV infection. We suggest that the HIV population should be considered a high-risk group and screened accordingly, with reduced intervals between pap smears, ultrasonography, or colonoscopy.

Our study has several strengths. Most importantly, the Taiwan NHI includes data from a longitudinal cohort and is a large and population-based database. The nationwide LHID 2000 provided an excellent resource for evaluating the risk of cancer in patients with HIV infection, so our study is valuable for establishing a cancer screening program for HIV-infected patients. Nevertheless, a number of limitations should be considered when interpreting our findings. First, HIV infection status is not indicated on health insurance cards, and a few patients with HIV infection have not been reported to the Taiwan Centers for Disease Control because of hospital shopping. Therefore, the data from the Centers for Disease Control on reported communicable diseases is not synchronized with the data set of the NHI program. We thought that the data set from the NHI program was more accurate because of those patients with HIV infection who have not been reported to the Taiwan Centers for Disease Control. And, the duration of HIV infection could be possibly underestimated in this study. Second, there was a lack of information about some risk behaviors, including personal habits, smoking, and lifestyle, which may not allow direct assessment of exposure to risk factors for known cancer. Third, there were no laboratory data on the CD4 cell count recorded in the NHI Research Database. Therefore, we could not determine how severe the HIV infections were in our study patients. The latest limitation, although we obtained 99.28% of the enrolled 1,115 HIV-infected patients had received ART, the percentage of HIV-infected patients receiving ART at the time of cancer diagnosis can not be obtained in this database*.*

## Conclusions

This study found that patients with HIV infection had a significantly higher risk of developing cancer compared with patients without HIV infection, especially breast cancer, oral cancer, colorectal cancer, liver cancer, respiratory and intrathoracic cancer, bone and connective tissue cancer, genitourinary cancer, lymphoma, and central nervous system cancer. Our results support an active and accelerated screening schedule in patients with HIV infection for breast, oral, colorectal, liver, and cervical cancer. However, patients with HIV infection should still undergo regular screenings for cancer based on the recommendations for the general population. Moreover, specific screening guidelines for cancer should be established for patients with HIV infection to increase their quality of life and life span.

### Ethics

Because the LHID data set consists of de-identified and secondary data released to the public for research, the study was exempt from full review by the Institutional Review Board of Taipei Medical University.

We can not get the informed consent from patients because this is a de-identified and secondary database.

## References

[1] Gotti D, Raffetti E, Albini L, Sighinolfi L, Maggiolo F, Di Filippo E (2014). Survival in HIV-infected patients after a cancer diagnosis in the cART Era: results of an Italian multicenter study. PLoS One.

[2] Hymes KB, Cheung T, Greene JB, Prose NS, Marcus A, Ballard H (1981). Kaposi’s sarcoma in homosexual men-a report of eight cases. Lancet.

[3] Curran JW, Jaffe HW (2011). AIDS: the early years and CDC’s response. MMWR Surveill Summ.

[4] Bouvard V, Baan R, Straif K, Grosse Y, Secretan B, El Ghissassi F (2009). WHO International Agency for Research on Cancer Monograph Working Group: A review of human carcinogens–Part B: biological agents. Lancet Oncol.

[5] Detels R, Muñoz A, McFarlane G, Kingsley LA, Margolick JB, Giorgi J (1998). Effectiveness of potent antiretroviral therapy on time to AIDS and death in men with known HIV infection duration. Multicenter AIDS Cohort Study Investigators. JAMA.

[6] Smith CJ, Ryom L, Weber R, Morlat P, Pradier C, Reiss P (2014). Trends in underlying causes of death in people with HIV from 1999 to 2011 (D:A:D): a multicohort collaboration. Lancet.

[7] Liu CY, Hung YT, Chuang YL, Chen YJ, Weng WS, Liu JS (2006). Incorporating development stratification of Taiwan townships into sampling design of large scale health interview survey. J Health Manag.

[8] Charlson ME, Pompei P, Ales KL, MacKenzie CR (1987). A new method of classifying prognostic comorbidity in longitudinal studies: development and validation. J Chronic Dis.

[9] Cooley TP (2003). Non–AIDS-defining cancer in HIV-infected people. Hematol Oncol Clin North Am.

[10] Intra M, Gentilini O, Brenelli F, Chagas EM, Veronesi U, Sandri MT (2005). Breast cancer among HIV infected patients: the experience of the European Institute of Oncology. J Surg Oncol.

[11] Goedert JJ, Schairer C, McNeel TS, Hessol NA, Rabkin CS, Engels EA; HIV/AIDS Cancer Match Study (2006). HIV/AIDS Cancer Match Study, Risk of breast, ovary, and uterine corpus cancers among 85,268 women with AIDS. Br J Cancer.

[12] Shiels MS, Cole SR, Kirk GD, Poole C (2009). A meta-analysis of the incidence of non-AIDS cancers in HIV-infected individuals. J Acquir Immune Defic Syndr.

[13] Sigel K, Dubrow R, Silverberg M, Crothers K, Braithwaite S, Justice A (2011). Cancer screening in patients infected with HIV. Curr HIV/AIDS Rep.

[14] Simard EP, Engels EA (2010). Cancer as a cause of death among people with AIDS in the United States. Clin Infect Dis.

[15] Shiels MS, Pfeiffer RM, Engels EA (2010). Age at cancer diagnosis among persons with AIDS in the United States. Ann Intern Med.

[16] Vogel M, Friedrich O, Lüchters G, Holleczek B, Wasmuth JC, Anadol E (2011). Cancer risk in HIV-infected individuals on HAART is largely attributed to oncogenic infections and state of immunocompetence. Eur J Med Res.

[17] Biggar RJ, Chaturvedi AK, Goedert JJ, Engels EA; HIV/AIDS Cancer Match Study (2007). AIDS-related cancer and severity of immunosuppression in persons with AIDS. J Natl Cancer Inst.

[18] Lin CH, Liau JY, Lu YS, Huang CS, Lee WC, Kuo KT (2009). Molecular subtypes of breast cancer emerging in young women in Taiwan: evidence for more than just westernization as a reason for the disease in Asia. Cancer Epidemiol Biomarkers Prev.

[19] Taiwan Breast Cancer Foundation: Current status of breast cancer in Taiwan. http://www.breastcf.org.tw/index.php/knowledge-base/current-status.

[20] Lin C, Chien SY, Chen LS, Kuo SJ, Chang TW, Chen DR (2009). Triple negative breast carcinoma is a prognostic factor in Taiwanese women. BMC Cancer.

[21] Grulich AE, Li Y, McDonald AM, Correll PK, Law MG, Kaldor JM (2001). Decreasing rates of Kaposi’s sarcoma and non-Hodgkin’s lymphoma in the era of potent combination anti-retroviral therapy. AIDS.

[22] Crum-Cianflone N, Hullsiek KH, Satter E, Marconi V, Weintrob A, Ganesan A (2009). Cutaneous malignancies among HIV-infected persons. Arch Intern Med.

[23] Parkin DM, Bray F, Ferlay J, Pisani P (2005). Global cancer statistics. CA Cancer J Clin.

[24] Shiels MS, Pfeiffer RM, Gail MH, Hall HI, Li J, Chaturvedi AK (2011). Cancer burden in the HIV-infected population in the United States. J Natl Cancer Inst.

[25] Mani D, Haigentz M, Aboulafia DM (2012). Lung cancer in HIV infection. Clin Lung Cancer.

[26] Clifford GM, Lise M, Franceschi S, Egger M, Bouchardy C, Korol D (2012). Lung cancer in the Swiss HIV Cohort Study: role of smoking, immunodeficiency and pulmonary infection. Br J Cancer.

[27] Bureau of Health Promotion, D.O.H., Taiwan, ROC (2010). Cancer Registry Annual Report, 1995–2008.

[28] Warnakulasuriya S (2009). Causes of oral cancer–an appraisal of controversies. Br Dent J.

[29] Palefsky J (2007). Human papillomavirus infection in HIV-infected persons. Top HIV Med.

[30] Chaiyachati K, Cinti SK, Kauffman CA, Riddell J (2008). HIV-infected patients with anal carcinoma who subsequently developed oral squamous cell carcinoma: report of 2 cases. Int Assoc Physicians AIDS Care (Chic).

[31] Guidleine for the Use of Antiretroviral Agents in HIV-1-Infected Adults and Adolescents in 2014. http://aidsinfo.nih.gov/guidelines.

[32] Chapman C, Aboulafia DM, Dezube BJ, Pantanowitz L (2009). Human immunodeficiency virus-associated adenocarcinoma of the colon: clinicopathologic findings and outcome. Clin Colorectal Cancer.

[33] Fattovich G, Stroffolini T, Zagni I, Donato F (2004). Hepatocellular carcinoma in cirrhosis: incidence and risk factors. Gastroenterology.

[34] Bodaghi S, Yamanegi K, Xiao SY, Da Costa M, Palefsky JM, Zheng ZM (2005). Colorectal papillomavirus infection in patients with colorectal cancer. Clin Cancer Res.

[35] Dalgleish AG, O’Byrne KJ (2002). Chronic immune activation and inflammation in the pathogenesis of AIDS and cancer. Adv Cancer Res.

[36] Bica I, McGovern B, Dhar R, Stone D, McGowan K, Scheib R (2001). Increasing mortality due to end-stage liver disease in patients with human immunodeficiency virus infection. Clin Infect Dis.

[37] Benhamou Y, Bochet M, Di Martino V, Charlotte F, Azria F, Coutellier A (1999). Liver fibrosis progression in human immunodeficiency virus and hepatitis C virus coinfected patients. The Multivirc Group Hepatology.

[38] Bräu N, Salvatore M, Ríos-Bedoya CF, Fernández-Carbia A, Paronetto F, Rodríguez-Orengo JF (2006). Slower fibrosis progression in HIV/HCV-coinfected patients with successful HIV suppression using antiretroviral therapy. J Hepatol.

[39] Silverberg MJ, Chao C, Leyden WA, Xu L, Tang B, Horberg MA (2009). HIV infection and the risk of cancers with and without a known infectious cause. AIDS.

